# Malaria Control and the Intensity of *Plasmodium falciparum* Transmission in Namibia 1969–1992

**DOI:** 10.1371/journal.pone.0063350

**Published:** 2013-05-07

**Authors:** Abdisalan M. Noor, Victor A. Alegana, Richard N. Kamwi, Clifford F. Hansford, Benson Ntomwa, Stark Katokele, Robert W. Snow

**Affiliations:** 1 Malaria Public Health Department, Kenya Medical Research Institute-Wellcome Trust Research Programme, Nairobi, Kenya; 2 Centre for Tropical Medicine, Nuffield Department of Clinical Medicine, University of Oxford, Oxford, United Kingdom; 3 Office of the Minister, Ministry of Health and Social Services, Windhoek, Namibia; 4 National Institute for Tropical Diseases, Tzaneen, South Africa; 5 National Vector-borne Diseases Control Programme, Directorate of Special Programmes, Ministry of Health and Social Services, Windhoek, Namibia; Tulane University School of Public Health and Tropical Medicine, United States of America

## Abstract

**Background:**

Historical evidence of the levels of intervention scale up and its relationships to changing malaria risks provides important contextual information for current ambitions to eliminate malaria in various regions of Africa today.

**Methods:**

Community-based *Plasmodium falciparum* prevalence data from 3,260 geo-coded time-space locations between 1969 and 1992 were assembled from archives covering an examination of 230,174 individuals located in northern Namibia. These data were standardized the age-range 2 to less than 10 years and used within a Bayesian model-based geo-statistical framework to examine the changes of malaria risk in the years 1969, 1974, 1979, 1984 and 1989 at 5×5 km spatial resolution. This changing risk was described against rainfall seasons and the wide-scale use of indoor-residual house-spraying and mass drug administration.

**Results:**

Most areas of Northern Namibia experienced low intensity transmission during a ten-year period of wide-scale control activities between 1969 and 1979. As control efforts waned, flooding occurred, drug resistance emerged and the war for independence intensified the spatial extent of moderate-to-high malaria transmission expanded reaching a peak in the late 1980s.

**Conclusions:**

Targeting vectors and parasite in northern Namibia was likely to have successfully sustained a situation of low intensity transmission, but unraveled quickly to a peak of transmission intensity following a sequence of events by the early 1990s.

## Introduction

Namibia is one of six sub-Saharan African countries that have formally declared ambitions to eliminate malaria following significant recent declines in clinical incidence [1]–[3]. Namibia has a long history of vector control and mass drug administration [4]–[5], however there has been no formal quantification of the epidemiology of malaria during this period to provide the context for current elimination ambitions. To understand the challenges to achieving the country’s ambition of malaria elimination by 2020 [2], lessons from the past are valuable to making rational planning decisions today. Evidence on the levels of interventions scale-up, the subsequent rate of epidemiological transition, and levels of rebound of risks where intervention may have been interrupted, all inform the feasibility of malaria elimination in Namibia.

Advances in computing and geostatistical techniques [6] and the increasing availability of data on malaria transmission have increased our ability to define the spatial and temporal risks of malaria endemicity at high spatial resolutions nationally [7]–[10] and globally [11], [12]. These maps, however, are currently available as predictions to contemporary time-points and do not provide information on the levels of historical risks that is required as the baseline upon which any potential transition of malaria risk is measured, and the probable rebound risks if control were interrupted are defined.

Here, we describe the scale and scope of control in Namibia from 1965 to 1992 in targeted areas in northern regions of the country and spatially model *Plasmodium falciparum* transmission intensity every 5 years using Bayesian goestatistics, from 1969 to 1989, to provide an epidemiological and control context and the likely impact of interventions during this period.

### Malaria Epidemiology and Control in Namibia

**Figure 1:**
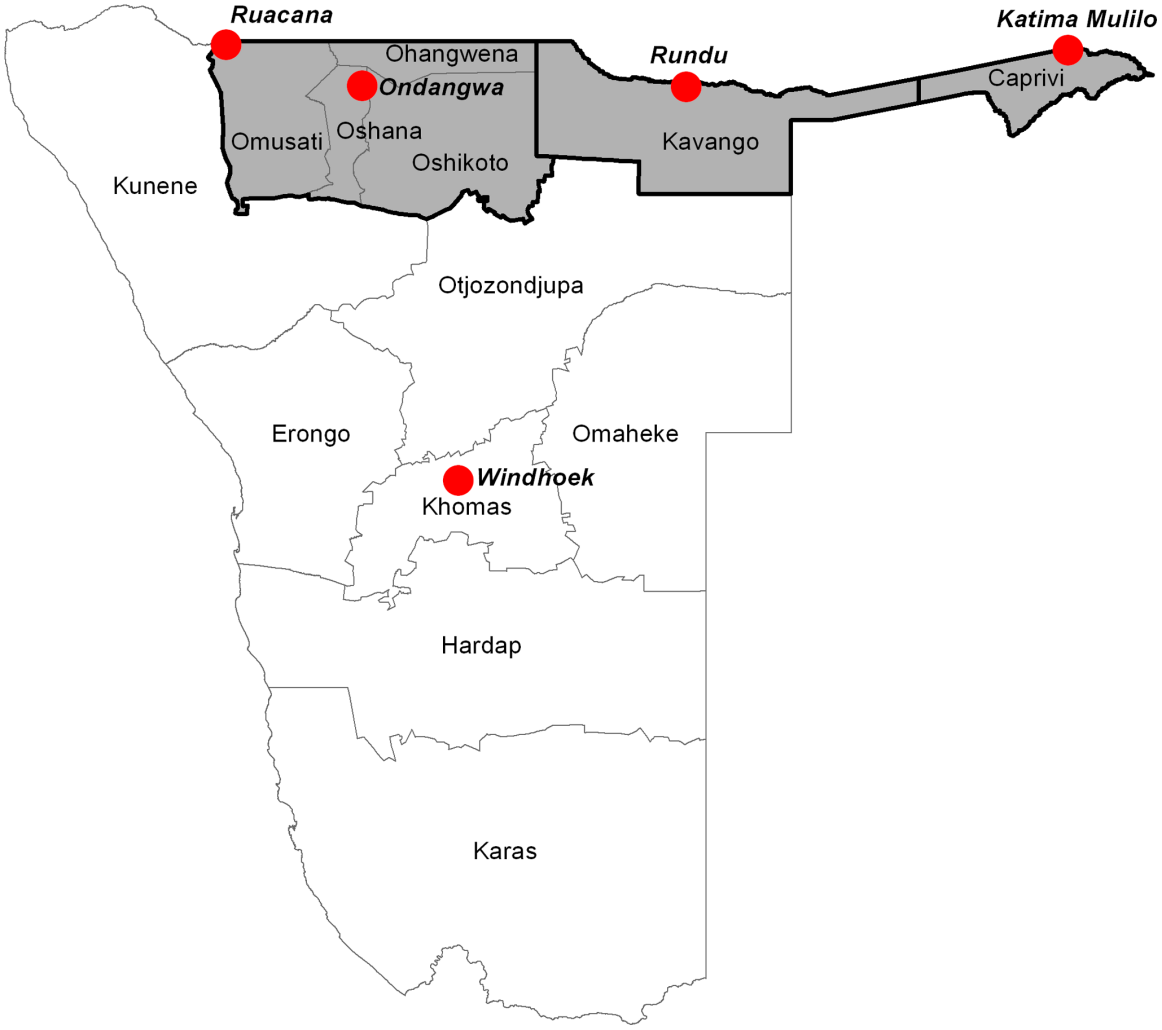
The boundaries of the 13 first level administrative units (regions) of Namibia and locations of the Capital City, Windhoek, the main towns of the north (Rundu, Katima Mulilo, Ruacana and Ondangwa). The grey shaded areas with thick black boundary are the intervention areas of Caprivi, Kavango and Ovambo.

The earliest surveys of malaria were undertaken in a non-random sample of communities across the country from February to June 1950 [13]. Information was collected on spleen and parasite rates and vector species to define zones of transmission across the country. The regions (Figure 1) of Ovambo (present day Omusati, Oshana, Ohangwena and Oshikoto regions), Kavango and Caprivi bordering Angola and parts of Bushmanland (part of present day eastern Otjozondjupa region) were defined as supporting intense, stable malaria transmission. The Southern regions from Erongo, Khomas and southern Omaheke to the Orange River on the border with South Africa were defined as largely free of transmission or with very focal pockets of occasional transmission [13]–[14].

Indoor residual spraying (IRS) using dichlorodiphenyltrichloroethane (DDT) started in 1965 in urban residential houses in the north of the country [4], [14], [15]. This was expanded to include all malarious areas in the northern territories following the appointment of a malaria public health specialist from South Africa to establish a network of malaria health inspectors in the areas of Ovambo and Kavango. The malaria programme for Ovambo and Kavango were managed from Windhoek with regional officers at Oshakati and Rundu respectively. Initially, the East Caprivi programme was supervised from Pietersburg but in 1967 was transferred to Pretoria in South Africa before returning to Pietersburg in 1972. Soon after, the numbers of field staff reduced and coverage with IRS deteriorated. Supervision for the East Caprivi was taken over from Windhoek in 1972 with a field station in Katima Mulilo.

**Figure 2:**
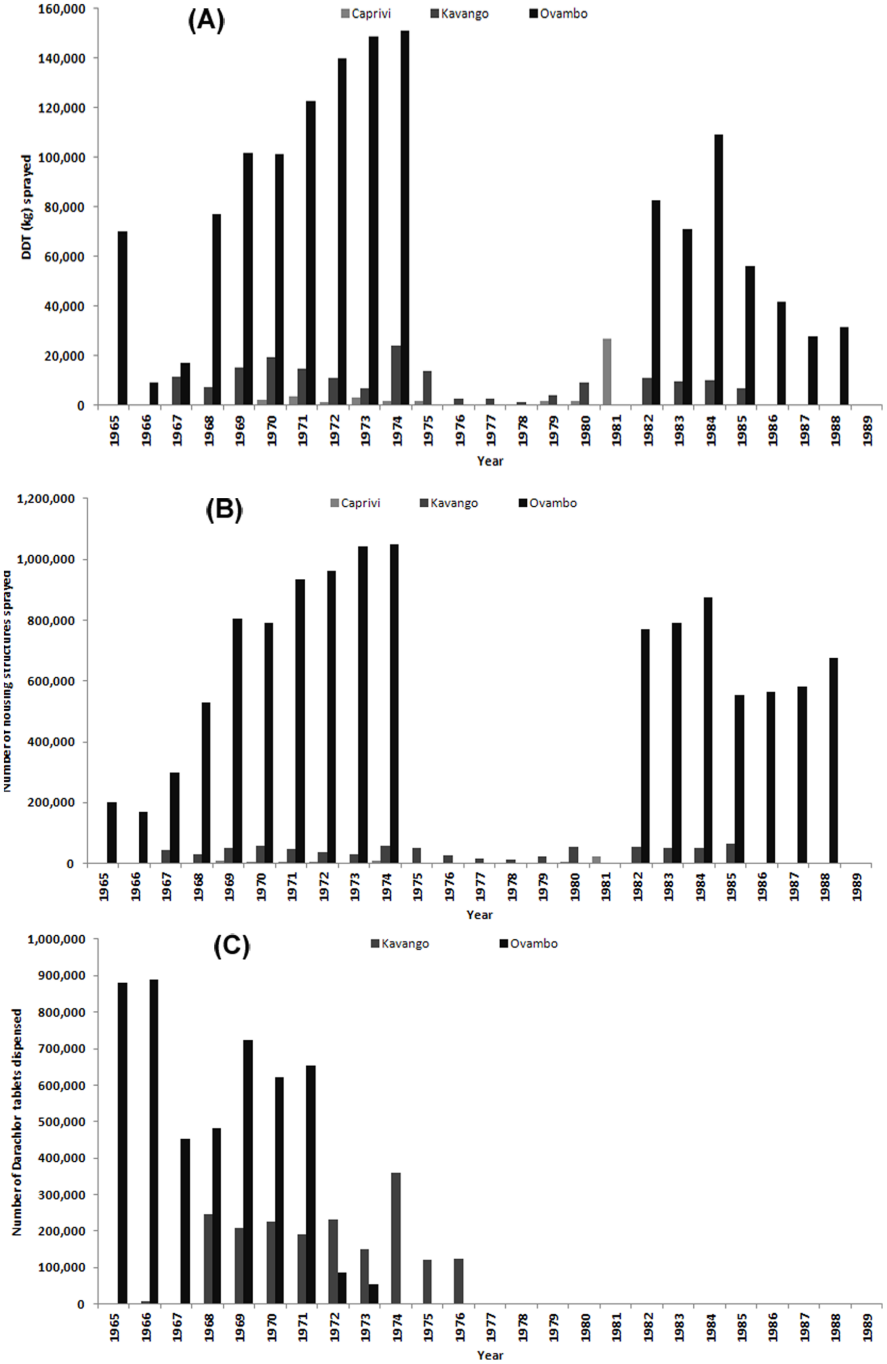
Summary intervention distribution and coverage data assembled from monthly and annual reports archived at Tzaneen for Caprivi, Kavango and Ovambo (present-day Omusati, Oshana, Ohangwena and Oshikoto regions) of: A) the amount of DDT (kilograms) used for IRS; B) the number of house structures sprayed; and C) the number of Darachlor tablets dispensed during the period 1965 to 1989 in A) Kavango and B) Ovambo. Data were unavailable for DDT, housing structures sprayed and Darachor tablets dispensed for the years where data is not shown. Darachlor use was terminated after 1989.

Spray operations were undertaken from May to November in Ovambo and twice annually (August to November and January to March) in Kavango and Caprivi [5]. Between 1966 and 1979, over 1.6 million kilograms of DDT was used to spray approximately 12.4 million housing structures in Ovambo, Kavango and Caprivi (Figure 2). In 1980 Bendiocarb was introduced to replace DDT in urban areas. From 1966, approximately 6.7 million tablets of Darachlor (chloroquine+pyrimethamine) were distributed (Figure 2) from January to June each year at dosages of 2 tablets for persons 10 years of age and above and 1 tablet to children below 10 years of age [5].

In 1966, annual mass blood surveys and treatment of febrile cases with Darachlor began in Ovambo and was extended to Kavango and Caprivi in 1967. Technical staff from the National Institute for Tropical Diseases (NITD) in Tzaneen, South Africa, were responsible for these parasitological surveys, which continued through to the late 1980s when large cross-sectional surveys were harder to undertake in part due to the insecurity during the war for independence. A malaria epidemic followed a reduction in both control and survey work in 1990 [16].

### Malaria Risk Mapping Process

#### Assembling *P. falciparum* parasite prevalence data

Community based *P. falciparum* parasite rate (*Pf*PR) survey data were compiled from monthly and annual reports available at the archives at the NITD in Tzaneen, South Africa, in 2011. It was possible to transcribe the data at village levels from summaries of the serial mass blood surveys during the years 1967 to 1992 across Ovambo, Kavango, Caprivi, Bushmanland (Otjozondjupa), Hereroland (Omaheke) and Damaraland (Kunene and parts of Erongo). Overall, communities and schools were visited each year at the end of the main transmission season usually between March and June to undertake cross-sectional surveys of infection prevalence. In the majority of surveys individuals of all ages were selected for malaria testing with a few surveys focusing only on children. Thick and thin Giemsa stained blood smears were taken from individuals and transported to the NITD where they were examined using light microscopy by expert microscopists before the results were returned to Namibia to support annual control planning. These active mass blood surveys were suspended after 1992. The longitude and latitude of all survey locations were identified using a variety of approaches including online village databases and gazetteers and a settlement database mapped using Global Positioning Systems (GPS) receivers [17]. Data were assembled as part of routine surveillance system for malaria control. Approval was provided by the Ministries of Health at the time. All data were de-identified and analysed anonymously.

#### Modelling historical *P. falciparum* risk within the limits of stable transmission

**Figure 3:**
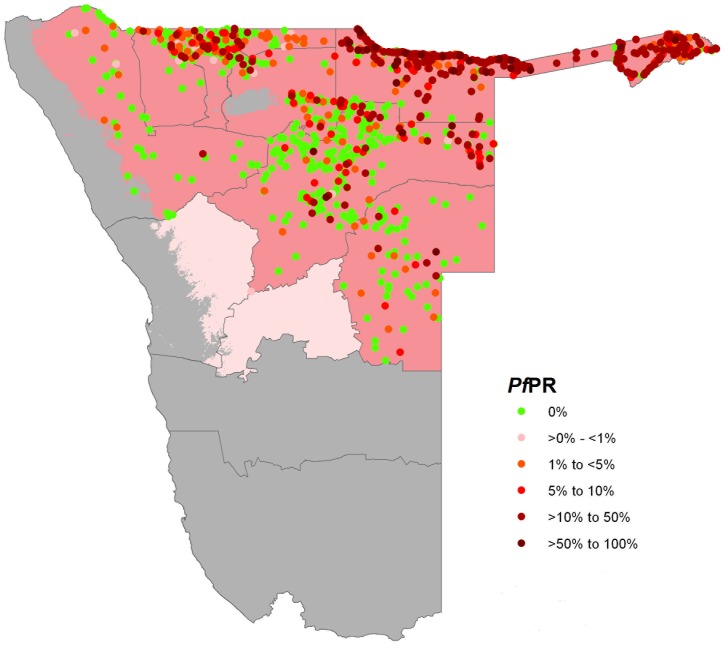
Map of contemporary first level administrative units of Namibia showing the spatial limits of P.falciparum transmission (dark pink) and location of PfPR survey data from the year 1967 to 1992. Where survey data are available for a location in more than one year, the highest PfPR value is displayed top. Light pink areas support unstable transmission and grey areas are malaria free. **Footnote:** A temperature suitability index (TSI) for malaria transmission at 1×1 km spatial resolution [10] was used to delineate areas in Namibia where malaria is unlikely to occur, defined as areas where TSI was equal to zero. TSI was constructed using monthly temperature time series within a biological modelling framework to quantify the effect of ambient temperature on sporogony and vector survivorship and determine the suitability of an area to support transmission globally separately for both P. falciparum and P. vivax. Extreme aridity was defined using synoptic mean monthly enhanced vegetation index (EVI) data [11] to classify into areas unlikely to support transmission, defined as areas where EVI was <0.1 in any two consecutive months of the year [7], [12]. In Namibia TSI and aridity identified the Namib Desert on the Atlantic Coast, parts of the Kalahari Desert in the South and the Etosha and other smaller saltpans as areas that were too hot or dry to support malaria transmission. Finally areas defined as operationally risk free based on reported incidences [2], [6], [7], [24] were identified (southern parts of the regions of Kunene and Omaheke and all of Erongo, Hardap, Khomas and Karas). It is hard to eliminate the possibility of any risks and therefore the parts of Erongo, the whole of Khomas and the southern parts of Omaheke were classified to be of unstable transmission where aridity and temperature do not exclude transmission.

Three previously described criteria [14] were used to define the limits of stable malaria transmission in Namibia. These were: the suitability of ambient temperature [18]; aridity [19]–[20]; and medical intelligence [14]. The resulting map classified areas in Namibia into areas that are unsuitable for transmission, those that support unstable transmission and areas of stable transmission (Figure 3, see footnote to this figure for details).

A standard set of ecological and climatic determinants were tested for association with the assembled *P. falciparum* prevalence data within the stable limits of transmission. These covariates were urbanisation [21], temperature suitability index (TSI) [18], annual average enhanced vegetation index (EVI) [22], annual average precipitation [19] and proximity to main water features [23]. The values of these covariates were extracted to each survey location using ArcGIS 10 Spatial Analyst (ESRI Inc. NY, USA) tool. A total-sets analysis based on a generalized linear regression model and implemented in *bestglm* package in R [24], [25] was then used to select those covariates that were most predictive of *P. falciparum* prevalence. The best combination of covariates, which was those with the lowest value of the Bayesian Information Criteria (BIC) statistic [26], was selected for the prediction of malaria risk.

A Bayesian model-based geostatistical (MBG) framework was used to produce continuous maps of P. falciparum prevalence at 5×5 km spatial resolution using data from 1967–1992. Individuals participating in each PfPR survey were assumed to be P. falciparum positive with a probability that was the product of a continuous function of the time and location of the survey and an age-correction factor derived from the age range of sampled individuals. A Gaussian random field [27] was used to model the continuous functions of time and space while the age-standardisation factors were modelled using a Bayesian version of the procedure described by [28] to prove predictions within a standard age range 2–10 years (*Pf*PR_2–10_).

Formally, each of the *N_i_* individuals in sample *i* at a location *x* and time *t* was taken to be *P. falciparum* positive with probability 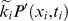, so that the number positive 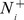 was binomially distributed as follows:
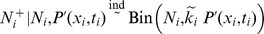
Where the coefficients 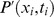 were modelled as a Gaussian process and the factor 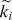 converted 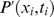 to the probability that individuals within the age range for study *i* at a location *x* and time *t* were detected *P. falciparum* positive, accounting for the influence of age on the probability of detection. The age-standardisation factor 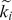 in each population was assumed drawn independently from a distribution 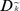 whose form is described in the Supplementary Information 1 (SI1), equation SI.2. Skew normal priors were assigned to the square root of the partial sill and the spatial range parameter. A vague but proper prior with an expectation of ten years was used for temporal scale parameter. Uniform priors were assigned to the direction of anisotropy parameter, the square of the “eccentricity” parameter which controls the amount of anisotropy and the temporal parameters for the amplitude of the sinusoidal component and the limiting autocorrelation in the temporal direction. Details of the model specifications are provided in SI1.

The MBG procedure began with an inference stage in which Monte Carlo Markov Chain (MCMC) simulation was used to fit both the geostatistical and age-correction models and a spatial-temporal prediction stage in which samples were generated from the posterior distribution of *Pf*PR_2–10_ at 5×5 km grid within the limits of stable transmission. The mid-point (in decimal years) between the recorded start and end months was used to temporally reference each survey. For each grid location samples of the annual mean of the full posterior distribution of *Pf*PR_2–10_ for the years 1969, 1974, 1979, 1984 and 1989 were generated. These were then combined to generate a single continuous map of mean *Pf*PR_2–10_. The annual mean *Pf*PR_2–10_ maps were classified into the endemicity classes of *Pf*PR_2–10_<1% (low stable endemic control [29]; *Pf*PR_2–10_ 1% - <5% (hypoendemic 1); *Pf*PR_2–10_ 5% - <10% (hypoendemic 2); PfPR_2–10_ 10% - ≤50% (mesoendemic); *Pf*PR_2–10_>50% (hyper- and holo-endemic).

Model accuracy was estimated by computing the linear correlation, the mean prediction error (MPE) and mean absolute prediction error (MAPE) of the observations and predictions of a 10% hold-out dataset. MPE is a measure of overall model bias while MAPE is a measure model accuracy. The hold-out set was selected using a spatially and temporally declustered algorithm [30] which defined Thiessen polygons around each survey location. Each data point had a probability of selection proportional to the area of its Thiessen polygon so that data located in densely surveyed regions had a lower probability of selection than those in sparsely surveyed regions setting a high threshold for model performance. The Bayesian spatio-temporal geostatistical model was then implemented in full using the remaining 90% of data and predictions were made to the 10% hold-out.

## Results

Data assembled comprised 3,497 community PfPR surveys from the period 1967 to 1992 of which 3,260 covering an examination of 230,174 individuals were successfully geocoded and subsequently used in the mapping of risk. The distribution of the PfPR data against the limits of transmission is shown Figure 3. All the tested environmental covariates were separately significant predictors of PfPR but taken together only EVI, precipitation and urbanisation were selected in the best-fit model for the 1967–1992 datasets and subsequently used in the *Pf*PR_2–10_ prediction models. The model MPE was 1.6% while the MAPE of 7.5% indicating good model precision but moderate model bias. The linear correlation of the predicted and observed hold-out set was 0.61.

**Figure 4:**
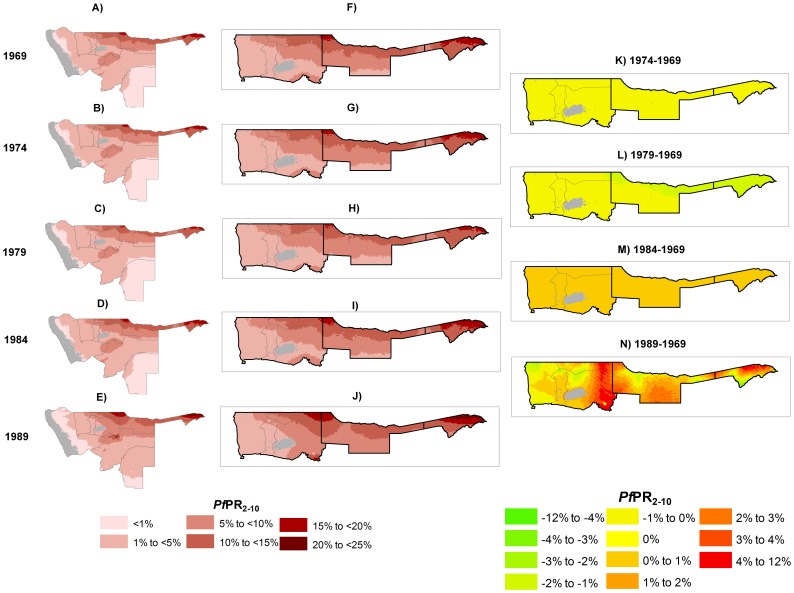
Panel A–D shows the posterior mean distribution of PfPR_2–10_ by the prediction years of 1969–1989 in Namibia. Panel F–J shows enlarged maps of the posterior mean distribution of PfPR_2–10_ by the prediction years of 1969–1989 in the three intervention regions of Caprivi, Kavango and Ovambo. Panel K–N shows the percentage difference from a reference year of 1969 of the PfPR_2–10_ predictions in 1974, 1979, 1984 and 1989. Negative/positive values indicate a decrease/increase in predicted PfPR_2–10_. The grey shade represents areas where transmission is unsuitable due to aridity. All maps are at 5×5 km spatial resolution.

The annual mean predictions of *Pf*PR_2–10_ reveal small to moderate overall reductions in transmission across the northern regions of Namibia over the period 1969 to 1979, followed by a small rise from 1984 with transmission reaching its maximum over this period by 1989 (Figure 4A–E). In 1969 the highest risk areas were concentrated in Caprivi, Ohangwena and northern Kavango where malaria transmission was largely mesoendemic (>10% to 25% *Pf*PR_2–10_). The southern half of Kavango, northern Oshikoto and an area at the confluence of Otjozondjupa, Oshikoto and Kunene regions had a predicted prevalence of 5% to 10% *Pf*PR_2–10_. Except for areas along the Namib Desert and almost all of Omaheke region where risks were <1% *Pf*PR_2–10_, the rest of country, within the limits of transmission, had predicted prevalence of 1% to <5% *Pf*PR_2–10_.

Between 1969 and 1974 the patterns of risk remained largely unchanged (Figure 4B) but by 1979 the extent of the higher risk classes of 5% to 10% and >10% to 50% *Pf*PR_2–10_ had reduced slightly (Figure 4C). In 1984, however, there appeared to be an expansion of the extent of risk with the high-risk areas (Figure 4D) expanding to margins greater than observed in 1969. This increase continued to 1989 in which the whole of Caprivi, most of Ohangwena and greater areas of northern Kavango were exposed to conditions supporting mesoendemic transmission. By 1989, most of Omaheke region, which was previously exposed to risks of <1% *Pf*PR_2–10_, experienced risks of >1% to <5% *Pf*PR_2–10_ (Figure 4E).

Focusing only on the main intervention areas of Caprivi, Kavango and Ovambo and using 1969 as a reference year, the reduction in risk by 1974 was less 1% overall and by 1979 was between 1% and 2% in the whole of Caprivi and northern Kavango while remaining <1% in the Ovambo and the rest of Kavango (Figure 4F–J). Compared to 1969, transmission had increased by up to about 4% *Pf*PR_2–10_ in Caprivi and most of Kavango and between 4% and 12% in western Ovambo by 1989. The highest mean annual predicted prevalence at anytime from 1969 to 1989 was in Caprivi of around 25% *Pf*PR_2–10_.

## Discussion

We have assembled one of the largest long-term, national historical series of malaria infection prevalence in Africa (Figure 3), from surveys undertaken throughout a period of aggressive control targeting both adult vectors and host infection. While data are incomplete it is clear that huge quantities of both DDT and antimalarial drugs were used to control malaria along the northern territories of present day Namibia from the late 1960s through the 1970s (Figure 2). During this period of escalating malaria control large amounts of infection prevalence data were collected to review progress. What was not available to the malaria departments 40 years ago was the ability to model and map changing risk using advanced space-time techniques that interpolate point prevalence data to unsampled locations to accurately estimate risk across a wider area.

We have modelled the changing patterns of malaria transmission intensity between 1969 and 1989 using the assembled data. A striking observation was that the extents of intense transmission declined only marginally throughout the first 5 years of the control efforts, but declined more significantly within the first 10 years by 1979. Thereafter, the margins of higher endemicity risk classes began to expand and by the late 1980s had reached a peak that might be considered comparable to the pre-intervention state (Figures 4 A–E). Focusing only on control priority regions of Caprivi, Kavango and Ovambo, by 1989, the average percentage increase of *Pf*PR_2–10_ from 1969 was between 4% and 12%, with the biggest rebound occurring in eastern Kavango and Caprivi (Figure 4 I–J).

It is not possible here to provide empirical attribution of changing malaria risk to intervention coverage, however, a number of factors may have contributed to a marginal initial decline in risk followed by an expansion to higher risk. First, while the malaria programme maintained concerted efforts to spray houses and provide presumptive treatment this did not cover every household in every region and some regions, notably Caprivi, accessibility was a major obstacle to scale up of interventions. Second, the war for independence, which started in earnest in 1975 and ended in 1988 hampered access in some areas and was associated with large cross-border population movement with Angola probably reducing the likely impact of control on the Namibia side. Third, despite a gradual decline in the use of Darachlor, chloroquine resistance emerged in 1984 and had began to lead to significant clinical failures by the early 1990s [4], [2], [14]. Forth this region is subject to climatic anomalies that lead to the occurrence of several droughts and above average rainfall across the surveillance period [31]. Finally, human population movement between the higher risk Angola and the northern border regions of Namibia may have played a role in increasing transmission, especially during the war for independence when Namibian fighters engaged South African troops from their Angola bases [32].

**Figure 5:**
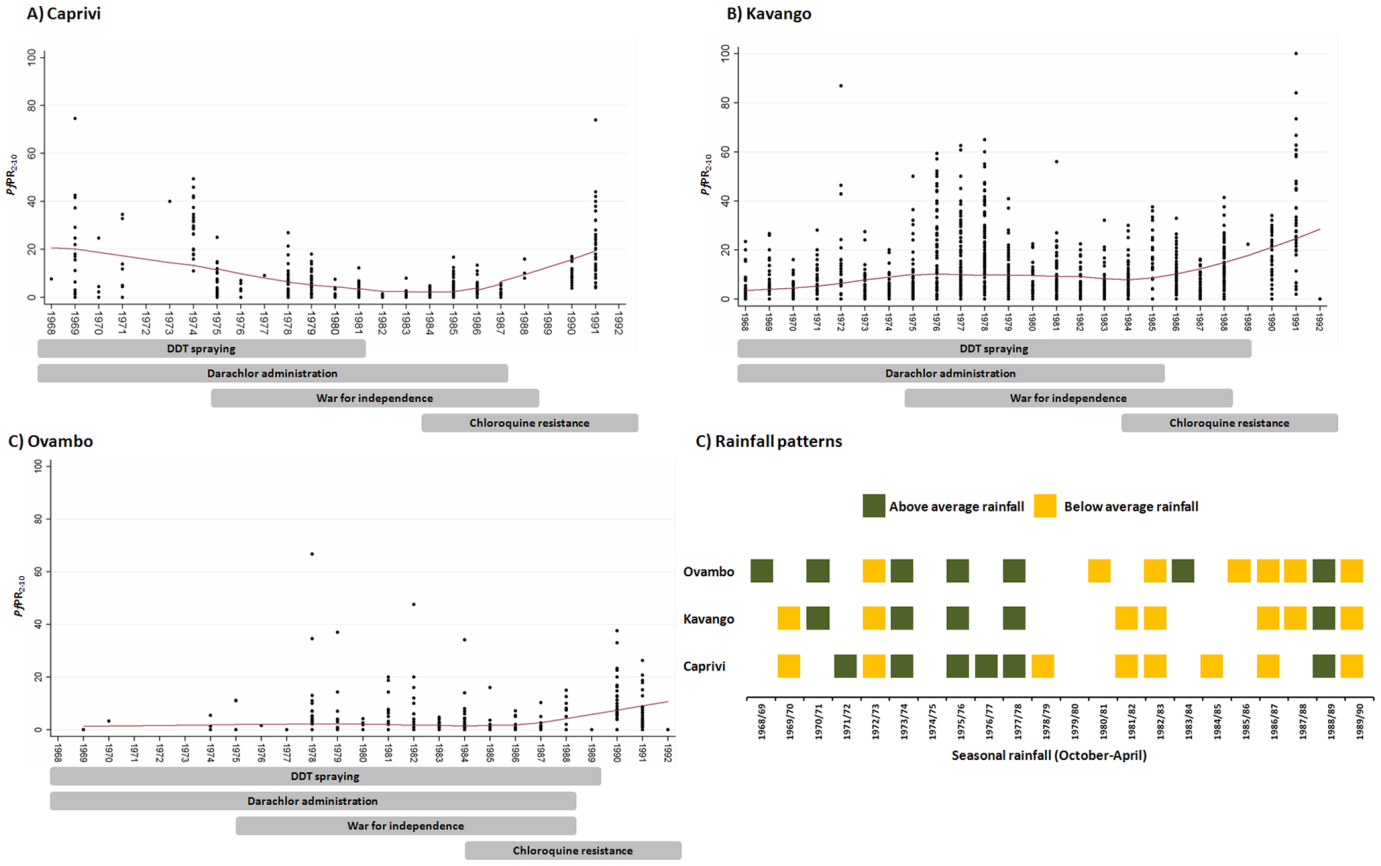
Graphs A–C show scatter plots with lowess regression fit of PfPR_2–10_ by year of survey and the main intervention and political events that may have determined the observed transmission patterns in Caprivi, Kavango and Ovambo respectively. D) is a summary of the droughts (orange) and above average rainfall (green) as measured using rainfall in the October – April rainfall in Caprivi, Kavango and Ovambo. Seasons not designated as one of drought or above average rainfall are considered to have received average rainfall.

To explore the plausibility of these events with changing patterns of observed *Pf*PR_2–10_, Figure 5A–D shows a lowess regression fit in each of the control regions of Caprivi, Kavango and Ovambo against some of the potential modulators of transmission. In Caprivi, the lowess fit of the observed data shows a starting average *Pf*PR_2–10_ of about 20% in 1969, declining to 5% in 1979 and 2% in 1984 before rebounding to 22% in 1989. The period of decline is coincident with the scale-up of DDT indoor residual spraying and Darachlor chemo-suppression. DDT spraying in Caprivi was stopped in 1981, several years earlier than in Kavango and Ovambo, but the decline in observed infection rates appeared to have continued through to 1985, perhaps because of the continued use of Darachlor. The summary of weather anomalies from the 1968/69 to the 1989/90 October-April rainfall season also shows that the first half of the surveillance years were dominated by periods of above average rainfall while in the second half there were more drought events in Caprivi. The year 1979 coincided with the drought of 1978/1979 that also occurred in the 1981/82, 1982/83 and 1984/85 seasons and may have contributed to the very low average infections rates in this region in both 1979 and 1984. In Kavango, average observed infection rates were at around 4% in 1969, rising and holding at about 10% from 1974 to 1979, reducing slightly to 8% in 1984 before rising from 1985 to the highest levels of 28% in 1989. Darachlor administration for chemo-suppression appears to have been stopped by 1985 although DDT spraying continued to 1989. Similar to Caprivi, above average rainfall events were common in the period 1969 to 1979 followed by frequent droughts thereafter. Coincident with the *Pf*PR_2–10_ prediction years also were droughts in the 1979/80 and the 1984/85 and extreme rainfall in the 1988/89 season. In Ovambo, average observed prevalence remained low throughout the period 1969 to 1986, at about 2%, before rising to approximately 10% by 1989. Both DDT spraying and Darachlor administration began earlier in Ovambo, several years before the surveillance period, and may explain the very low starting prevalence, although data before 1978 were sparse. DDT spraying and Darachlor administration continued to 1989 and 1988 respectively. The rainfall patterns over the surveillance period were similar to those of Caprivi and Kavango.

In the intervention areas, the years of above average rainfall generally coincided with much higher upper limits of observed *P. falciparum* infections compared to the years when a drought occurred although this seemed not to dramatically shift the average infections rates (Figure 5). In addition, despite the 13-year period of war starting 1975, a rise in infection rates seems to start only after 1984, although anecdotal information shows that the effect of the war on control may have started earlier. For example in western Caprivi, insecurity affected control efforts during the period 1979 to 1981. Independence fighters stationed in northern Angola also frequently crossed the border into Namibia carrying infections that may have led to localised outbreaks. In parts of Ovambo and Kavango, there was increased reluctance by residents to have their houses sprayed. The emergence of chloroquine resistance in 1984 and its gradual increase through to the 1990s may also have contributed to the observed rise in infection rates during this period across the three intervention areas and the subsequent reported epidemic of 1990 [33].

The intensive efforts and vector control using DDT and parasite suppression using Darachlor in Caprivi, Kavango and Ovambo appeared to be associated, at a glance, with only small to moderate reductions in transmission over the first 15 years of the surveillance period. It is not possible to define a pre-intervention, pre-1960s, endemicity across northern Namibia, although this is likely to have been considerably higher than the data shown for 1969 [13]. A reasonable description of the outcome of sustained control between 1969 and 1992 is that up to 1979 a combination of vector and parasite control measures, maintained transmission at a low level, probably negating the effect of the several periods above average rainfall. In the latter years despite declining control the frequent droughts probably helped sustain risks at low levels until the escalating war, the failure of chloroquine and the high rainfall of the 1988/89 season led to the epidemics of 1990 resulting in the expansion of high transmission across the northern territories of Namibia.

Following the epidemic in 1990, the National Malaria Control Programme, later renamed the National Vector-borne Disease Control Programme (NVDCP) was set up by the Namibian government [16]. In March 2009, a meeting of the Elimination Eight (E8) countries of the Southern Africa Development Community (SADC) was held in Windhoek with the aim of eliminating malaria in the four first tier countries (Botswana, Namibia, South Africa and Swaziland) by 2015 while accelerating control with the aim of eventual elimination in the four second tier countries (Angola, Mozambique, Zambia and Zimbabwe) [1]. In April 2010, the Namibian government launched a malaria elimination campaign to move the country to pre-elimination/elimination in the next five to ten years [34]. Human population movement has been identified as one of the major obstacles to malaria elimination and the historical maps of malaria risk developed in this study will contribute to the quantification of these risks as they describe the ‘probable’ innate transmission intensity in the malarious northern regions of Namibia.

In conclusion, the documented efforts directed at controlling malaria in northern Namibia probably sustained a period of low parasite transmission for over ten years, but the combination of climatic anomalies, insecurity, cross-border movement and a declining efficacy and coverage demonstrate how fragile control effects are and when these, harder to manage features occur, malaria transmission increases in its intensity and its spatial extent.

## Supporting Information

Information S1 **Model-based Geostatistical Procedures.**
(DOCX)
