# Supplementary material for: Malaria Control and the Intensity of Plasmodium falciparum Transmission in Namibia 1969–1992
Source: PLoS One. 2013 May 7;8(5):e63350. doi: 10.1371/journal.pone.0063350 (PMC3646760; doi:10.1371/journal.pone.0063350)
Supplement: Information S1 — Model-based Geostatistical Procedures. (DOCX) [file pone.0063350.s001.docx]

**Supplementary Information (SI) 1: Model-based Geostatistical Procedures**

Here we present the details of the MBG framework used to develop the malaria risk maps for Namibia. The MBG procedure was adopted from Gething et al 2011^1^ where further model details, especially on age-standardisation of parasite rate data, can also be found. The generic model code is available on Malaria Atlas Project *P. falciparum* Cartographic code link^2^ and has been adapted for Namibia. Model fitting was achieved using Markov chain Monte Carlo (MCMC)^3,4^.

**SI 1.1 The MBG presentation**

Each of the individuals in sample was assumed *P. falciparum* positive with probability , so the number positive was distributed binomially:

S1.1

The coefficient was modelled as a Gaussian process. The factor converted to the probability that individuals within the age range reported for study were *P. falciparum* positive, and that the infection was detected, thereby accounting for the influence of age on the probability of detection^5^. The age-standardisation factor in each population was assumed drawn independently from a distribution whose parameters were the lower and upper ages reported in study :

S1.2

The form of is described in Gething et al 2011^2^.

*Pf*PR_2-10_ is the *P. falciparum* parasite rate for individuals between ages 2 (2.00) and 10 (9.99). Its value at an arbitrary location and time is the product of and another age-standardisation factor, , distributed as :

S1.3

The factor converted to the probability that individuals between ages 2 and 10 at location are *P. falciparum* positive. The age-standardisation factor of a survey is the product of the age-standardisation factor associated with the same place, time and age range and the sensitivity of the survey.

The coefficient at arbitrary location and time was modelled as the inverse-logit function applied to a random field evaluated at , plus an unstructured (random) component .

S1.4

The components were assumed independent and identically distributed for each location and time and a standard diffuse but proper prior with expectation 0.25 was assigned to their variance .

S1.5

$\frac{1}{V}\sim Gamma(3,12)$ S1.6

The random field was modelled as a Gaussian process characterised by its mean and covariance functions:

S1.7

The mean function was defined as ,where was a vector consisting of a constant and *n* = 2 environmental covariates indexed by spatial location ,and was a corresponding vector of regression coefficients. The covariance of the field was modelled using a version of the spatiotemporal covariance function recently recommended by Stein^6^ (equation 2.12):

S1.8

is the modified Bessel function of the second kind of order , and is the gamma function [[7](#_ENREF_7),[8](#_ENREF_8)].

Spatial distance between a pair of points and was computed as great-circle distance multiplied by a factor that depends on the angle of inclination of the vector pointing from to . was computed as if latitude and longitude were Euclidean coordinates (on a cylindrical projection) to allow for anisotropy:

S1.9

When (that is, for points at the same location but different times), the covariance function reduces to

S1.10

As temporal separation increases, the covariance approaches a limiting sinusoid rather than zero. When , on the other hand (for points at different locations but the same time), it reduces to a standard exponential form with range parameter . Unlike standard sum-product models, this covariance function does not have problematic ridges along its axes^6^.

**SI 1.2 Prior Specification**

The square root of the partial sill and the spatial range parameter were assigned skew-normal priors:

S1.11

S1.12

and their specification is described further below.

The standard “one-over-*x*” prior for the temporal scale parameter resulted in collapse to zero, a common artefact when data do not contain strong information. A relatively vague but proper prior, which has an expectation of ten years, was used instead.

S1.13

A uniform prior was assigned to the direction of anisotropy parameter and to the square of the “eccentricity” parameter , which controls the amount of anisotropy,

S1.14

S1.15

a uniform prior was assigned to the temporal parameters governing the amplitude of the sinusoidal component and the limiting autocorrelation in the temporal direction :

S1.16

and a standard prior was assigned to the components of the mean:

S1.17

Although standard priors such as the improper “flat” prior^3^ were assigned to most of the basic model parameters, subjective skew-normal priors^7^ were specified for the range and partial sill parameters and .

**SI 1.3 Model implementation**

***SI 1.3.1 MCMC Algorithms***

Both the main geostatistical model and the age-standardisation sub-model were fitted using the MCMC algorithm^3,4^. The algorithm was implemented in the Python^8^ and FORTRAN programming languages using the open-source Bayesian statistics package PyMC^9,10^ and the numerical packages SciPy and NumPy^11^.

The evaluation of at the sampling locations and times was updated using Gibbs steps^3^. The evaluation of the uncorrelated process was updated one point at a time using random-walk Metropolis steps^3^. The model parameters , , , , , , and were updated jointly using the method of Haario, Saksman and Tamminen^12^.

Within the MCMC loop, the age-standardisation factors were not imputed explicitly. We were not interested in their particular values, and marginalizing out ”nuisance parameters” ahead of time usually improves the mixing of MCMC algorithms. Before the MCMC loop began, the marginal likelihood:

S1.18

was approximated using standard Monte Carlo integration for several values of . That is, values for the model parameters , , and and the age distribution were drawn from their posterior predictive distributions, then expression (S1.3) was evaluated to obtain , then the binomial probability was evaluated for several values of . The probabilities resulting from many such draws were averaged. Inside the MCMC loop, the marginal likelihood function for arbitrary values of was evaluated by interpolation.

***SI 1.3.2 Age Correction Model***

The age distribution parameters , and are independent of the relative *Pf*PR parameters , , , , , , and given the data, so these two groups of parameters were inferred using separate MCMC algorithms.

In the MCMC for the age distribution parameters, the survey populations’ age distributions were updated using Gibbs steps^3^. The concentration parameter was updated using random-walk Metropolis steps^3^. The typical age distribution was represented as a normalized sequence of gamma random variables^13^, and these variables were updated one at a time using random-walk Metropolis steps^3^.

In the MCMC for the relative *Pf*PR parameters, the distributional parameters , and were updated jointly using the method of Haario, Saksman and Tamminen^12^. The parameters , , , and were updated jointly for each population using the same method.

***SI 1.3.3 Spatiotemporal Prediction***

The output of the MCMC stage consisted of samples from the posterior of the parameter set and a corresponding samples from the posterior of the space-time random field at each of the data locations. For every sample, the conditional distribution of the annual mean of the space-time random field was predicted at each prediction location on the nodes of a regular 1×1 km grid within the spatial limits of stable *P. falciparum* transmission^14^. The distribution of the annual mean for prediction location was modelled as the joint multivariate normal distribution of the 12 predicted monthly values e.g $\left\{ t=1969jan, \ldots,{1969}_{Dec} \right\}$ for that year specified by a 12 element mean vector and 12 × 12 variance-covariance matrix :

S1.19

The mean vector was computed using:

S1.20

where and were the predicted mean of the random field at each of the 12 prediction times $\left\{ t={1969}_{Jan}, \ldots,{1969}_{Dec} \right\}$ at spatial location and at each of the data locations respectively, and were the data-to-prediction and data-to-data covariance matrices respectively, and was the vector of data values. The 12 × 12 variance-covariance matrix was computed using:

S.1.21

The value of the sample of , the variance of the unstructured component , was then added to the diagonal of the matrix and 1000 draws were made randomly from the distribution specified in equation S1.19 These draws represented samples from the posterior distribution of and were subject to an inverse logit transform and then multiplied by the sample of the age-standardisation parameter to form the sample from the posterior distribution of the predicted mean annual 1969, 1974, 1975, 1980, 1985 and 2010 *Pf*PR_2-10_ endemicity surfaces at location :

S.1.22

This procedure was repeated for every sample to form the set of samples for each prediction location. The point estimate of *Pf*PR_2-10_ endemicity at each location was defined as the mean of this set, whilst the probability of membership to each class was computed as the proportion of these samples falling within each class definition.

**References**

1. Gething PW, Patil AP, Smith DL, *et al*, 2011. A new world malaria map: Plasmodium falciparum endemicity in 2010. *Malar J 10*: 378 doi: 10.1186/1475-2875-10-378.
2. Malaria Atlas Project *P. falciparum* Cartographic code. https://github.com/malaria-atlas-project/mbgw-clean. Accessed December 2011.
3. Gilks WR, Spiegelhalter DJ, 1999. Markov Chain Monte Carlo in practice. Interdisciplinary Statistics. Boca Raton, Florida, U.S.A.: Chapman & Hall / CRC Press LLC.
4. Gelman A, Carlin JB, Stern HS, 1993. Bayesian data analysis. Texts in Statistical Science. Boca Raton, Florida, U.S.A.: Chapman & Hall / CRC Press LLC. 696 p.
5. Smith DL, Guerra CA, Snow RW, Hay SI, 2007. Standardizing estimates of the *Plasmodium falciparum* parasite rate. *Malar J 6*: 131.
6. Stein ML, 2005. Space-time covariance functions. *J Am Stat Assoc 100*: 310-321.
7. Azzalini A, 1985. A class of distributions which includes the normal ones. *Scand J Stat 12*: 171-178.
8. van Rossum G, 2008. Python Programming Language - Official Website. Website: URL http://www.python.org.
9. Fonnesbeck C, Huard D, Patil AP. PyMC 2.0 User’s Guide: installation and tutorial 2008. URL: http://www.trichech.us/pymc.
10. Patil A, Huard D, Fonnesbeck CJ, 2010. PyMC: Bayesian stochastic modelling in Python. *J Stat Softw* *35*: e1000301.
11. Oliphant TE, 2007. Python for scientific computing. *Comput Sci Eng 9*: 10-20.
12. Haario H, Saksman E, Tamminen J, 2001. An adaptive Metropolis algorithm. *Bernoulli 7*: 223-242.
13. Hogg RV, Craig AT, 2005. *Introduction to Mathematical Statistics*. Upper Saddle River, New Jersey, U.S.A: Prentice Hall Inc. 564 p.
14. Guerra CA, Gikandi PW, Tatem AJ, Noor AM, Smith DL, *et al,* 2008. The limits and intensity of *Plasmodium falciparum* transmission: implications for malaria control and elimination worldwide. *PLoS Med 5*: e38.
